# Heartburn’s Hidden Impact: A Narrative Review Exploring Gastroesophageal Reflux Disease (GERD) as a Cardiovascular Disease Risk Factor

**DOI:** 10.3390/jcm12237400

**Published:** 2023-11-29

**Authors:** Jacob J. Gries, Bing Chen, Salim S. Virani, Hafeez Ul Hassan Virk, Hani Jneid, Chayakrit Krittanawong

**Affiliations:** 1 Department of Internal Medicine, Geisinger Medical Center, Danville, PA 17822, USA; jgries1@geisinger.edu; 2 Department of Gastroenterology and Hepatology, Geisinger Medical Center, Danville, PA 17822, USA; 3 Section of Cardiology and Cardiovascular Research, Department of Medicine, Baylor College of Medicine, Houston, TX 77030, USA; 4 Office of the Vice Provost (Research), The Aga Khan University, Karachi 74800, Pakistan; 5 Harrington Heart & Vascular Institute, Case Western Reserve University, University Hospitals Cleveland Medical Center, Cleveland, OH 44106, USA; 6 Division of Cardiology, University of Texas Medical Branch, Houston, TX 77002, USA; 7 Cardiology Division, NYU Langone Health, NYU School of Medicine, New York, NY 10016, USA; 8 Cardiology Division, Section of Cardiology, NYU School of Medicine, 550 First Avenue, New York, NY 10016, USA

**Keywords:** gastroesophageal reflux disease (GERD), cardiovascular disease (CVD), coronary artery disease (CAD), atherosclerosis, myocardial infarction (MI), proton pump inhibitor (PPI)

## Abstract

Gastroesophageal reflux disease (GERD) is a very common disease with an estimated 442 million cases worldwide. It is a well-documented independent risk factor for many gastrointestinal pathologies, however, its role in cardiovascular disease (CVD) is unclear, despite its high prevalence in patients with CVD. Although traditionally considered a causative agent of noncardiac chest pain, a common imitator of cardiac chest pain, or an incidentally shared comorbidity in patients with CVD, a number of studies have implicated GERD and its therapies as risk factors for CVD. This narrative review will explore the relationship between GERD and CVD, including medical and mechanical therapeutic approaches for GERD that could potentially impact the incidence, progression, and mortality of CVD.

## 1. Introduction

Gastroesophageal reflux disease (GERD) is a very common disease with an estimated 442 million cases worldwide [1]. Its hallmark symptoms include heartburn and regurgitation that are frequently managed through use of acid-suppressing medications, particularly proton pump inhibitors (PPI) and histamine 2 receptor antagonists (H2RA). Refluxed acid through a compromised lower esophageal sphincter can produce burning chest pain that can be misinterpreted by patients and clinicians alike as cardiac chest pain, prompting potentially unnecessary cardiac workups. Despite this, a number of studies have implicated GERD as a risk factor for the onset and progression of cardiovascular disease (CVD) and not just a benign comorbidity. This is particularly important as cardiovascular disease remains the leading cause of mortality worldwide [2] and a poor diagnosis and management of GERD, a previously unassumed condition, may be contributing to its lethality.

This narrative review will explore potential pathophysiological mechanisms and therapies of GERD as a risk factor for the onset, progression, and adverse outcomes of CVD. It will also review the common presentations, current recommended diagnostic workup, as well as common medical and mechanical interventions of GERD, with hopes to reduce its symptomatic prevalence and, therefore, reduce the effect of GERD on CVD.

## 2. Materials and Methods

**Figure 1:**
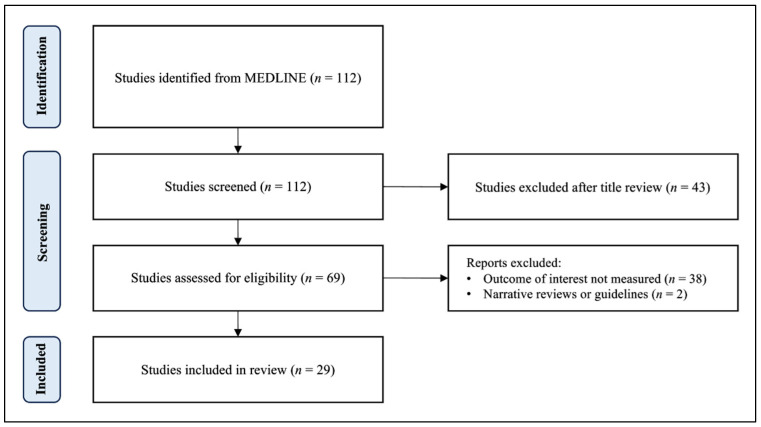
A flow diagram of study selection.

To identify relevant studies, a comprehensive search of the Pubmed/MEDLINE database was conducted to retrieve relevant articles from inception to September 2023. A combination of Medical Subject Heading (MeSH) terms and text words related to GERD and cardiovascular disease were used. MeSH terms included “gastroesophageal reflux disease”, “GERD”, “acid reflux”, “cardiovascular disease”, and “cardiac event”. Only English-language literature involving human subjects was included, and a range of study types, including prospective cohort studies, experimental studies, population studies, meta-analyses, umbrella reviews, retrospective cohort studies, clinical trials, and observational studies were eligible for inclusion. After screening and data extraction (Figure 1), the authors conducted a narrative synthesis of the studies, with the extracted data being summarized into tables for easy comparison and review.

## 3. Results and Discussion

The key studies on GERD or GERD therapy and its effects on cardiovascular disease are listed in Table 1.

### 3.1. Association between GERD and Coronary Atherosclerosis

A large population-based, questionnaire-based, cross-sectional, case-control study conducted by Jansson et al. in 2008 identified 3153 people with severe reflux symptoms against 40,210 controls [11]. Their initial findings suggested positive associations between GERD and acute myocardial infarction (AMI) (OR 1.7, 95% CI 1.4–2.1, *p*-value < 0.0001), angina pectoris (OR 2.5, 95% CI 2.1–2.9, *p*-value < 0.0001), and stroke (OR 1.6, 95% CI 1.2–2.1, *p*-value 0.0004) [2]. Only angina pectoris (OR 1.9, 95% CI 1.6–2.2, *p*-value < 0.0001) remained statistically significant when adjusted for age, sex, smoking, BMI, and socioeconomic factors [2]. Stroke (OR 1.3, 95% CI 1.0–1.7, *p*-value 0.08) and myocardial infarction (OR 1.2, 95% CI 1.0–1.5, *p*-value 0.05) demonstrated a diminished positive association after these adjustments but were not statistically significant [2]. This study suggests that a significant connection between GERD and myocardial infarction, angina pectoralis, and stroke may exist [2]. Another large prospective cohort study by Chen et al. in 2023 found that any gastrointestinal disease was associated with an elevated risk of overall CVD (HR 1.38, 95% CI 1.35–1.42) [12]. GERD was among the gastrointestinal diseases with the highest association (HR 1.41, 95% CI 1.35–1.46), with a stronger correlation in women, patients with a body mass index ≥25 kg/m^2^, and patients ≤60 years old [11].

A retrospective cohort study by Song et al. in 2022 identified 16,616 patients who underwent both an upper gastrointestinal endoscopy between 2003 and 2017 and a cardiac computed tomography (CT) within one year [13]. The degree of coronary atherosclerosis was measured using the coronary artery calcium scores (CACS). The results demonstrated higher CACSs (≥100) were present in patients with GERD (*p* = 0.008), but a high CACS did not increase the risk of GERD (OR = 1.007, 95% CI 0.857–1.182), nor did GERD increase the risk of a high CACS (OR = 1.018, 95% CI 0.865–1.198) [13]. Risk factors associated with a high CACS in patients with GERD include age (OR = 1.087, 95% CI 1.066–1.109), male sex (OR = 5.645, 95% CI 2.561–12.446), hypertension (OR = 1.800, 95% CI 1.325–2.446), and hypercholesterolemia (OR = 1.684, 95% CI 1.213–2.338) [13]. Another large prospective cohort study by Chen et al. in 2016 to determine the association between GERD and subsequent coronary artery disease using long-term use of PPIs as the independent variable [14]. A total of 12,960 patients who met these criteria between the years 2000 and 2011 were compared against 51,840 age, sex, and index year-matched controls. Both cohorts were followed up until the end of 2011 to determine the incidence of coronary artery disease [14]. Patients with GERD were found to have a greater probability of developing coronary artery disease [14]. After adjustment for age, sex, hypertension, diabetes, hyperlipidemia, alcohol-related disease, stroke, chronic obstructive pulmonary disease, asthma, biliary stone, anxiety, depression, chronic kidney disease, and cirrhosis (adjusted hazard ratio 1.49, 95% CI 1.34–1.66) [14]. The risk of coronary artery disease was even greater for patients treated with proton pump inhibitors for more than 1 year than for those treated with PPIs for less than 1 year [14]. This study further implicates GERD as an independent risk factor for developing cardiovascular disease and suggests that acid-suppressive therapy with PPIs also plays a role in the onset of coronary artery disease [14]. A Mendelian randomization study by Sun et al. in 2022 used summary statistics from genome-wide association studies for GERD and the FinnGen consortium for cardiovascular diseases to further analyze causal associations between GERD and 10 CVD outcomes as well as 14 cardiovascular risk factors [15]. Two-sample multivariable Mendelian randomization and mediation analysis were performed on the extracted data [15]. The results indicated that GERD was positively associated with seven CVD outcomes including coronary artery disease (OR 1.26, 95% CI 1.15–1.37), myocardial infarction (OR 1.41, 95% CI 1.28–1.57), atrial fibrillation (OR 1.34, 95% CI 1.19–1.51), heart failure (OR 1.34, 95% CI 1.21–1.50), any stroke (OR 1.30, 95% CI 1.18–1.43), ischemic stroke (OR 1.19, 95% CI 1.06–1.34), and venous thromboembolism (OR 1.29, 95% CI 1.16–1.44) [15]. Additionally, GERD was associated with cardiovascular risk factors, including BMI, HDL-C, triglyceride levels, type 2 diabetes mellitus, insomnia, and depression [15].

### 3.2. Association between GERD and Myocardial Ischemia

The resemblance between acute severe esophageal symptoms and acute anginal pain has frequently caused confusion among both patients and clinicians, sometimes leading to misinterpretation of symptoms as acute coronary syndrome [16]. Because of this, many patients have undergone cardiac stress tests or coronary angiographies that ultimately are suggestive of alternative noncardiac etiologies.

Nonetheless, the pathophysiology of GERD suggests that inflammatory cytokines are commonly elevated in GERD, including Il-1, IL-6, and platelet-activating factor, as well as the formation of reactive oxidative species, which can accelerate the progression of previous atherosclerotic lesions and precipitate acute coronary syndrome through plaque rupture [17]. Inflammatory cytokines may also play a role in the formation of acute thrombosis within the coronary arteries and other vessels in the cardiovascular system [17]. A 2020 study by Eisa et al. supported the elevated risk by comparing a large cohort of patients with GERD to those without GERD. The findings revealed a significantly increased occurrence of AMI in the GERD population compared to the non-GERD cohort (OR 1.11, 95% CI 1.08–1.13) [18].

A large population-based study by Lei et al. in 2017 compared the incidence of AMI in a cohort of 54,422 patients with newly diagnosed GERD with 269,572 matched controls without GERD [19]. A total of 1236 (0.5%) of the control patients and 371 (0.7%) of patients with GERD experienced an AMI during the mean follow-up period of 3.3 years [19]. GERD was independently associated with increased onset of AMI via Cox-proportional-hazard model analysis (HR = 1.48; 95% CI: 1.31–1.66, *p* < 0.001) [19]. An experimental study by Teragawa et al. in 2019 enrolled 236 patients with GERD and suspected coronary artery disease [20]. Each of the patients underwent coronary angiography and was divided into three cohorts based on the results: (1) organic coronary artery disease (>50% stenosis with ischemic findings, *n* = 141); (2) vasospastic angina (positive spasm provocation test without organic coronary artery disease, *n* = 52); and (3) no organic coronary artery disease or vasospastic angina [20]. Logistic regression analysis demonstrated that a history of GERD was associated with an increased incidence of vasospastic angina (OR 7.8; *p* < 0.01) [20].

### 3.3. Pathophysiology of GERD as a Risk Factor for CVD

**Figure 2:**
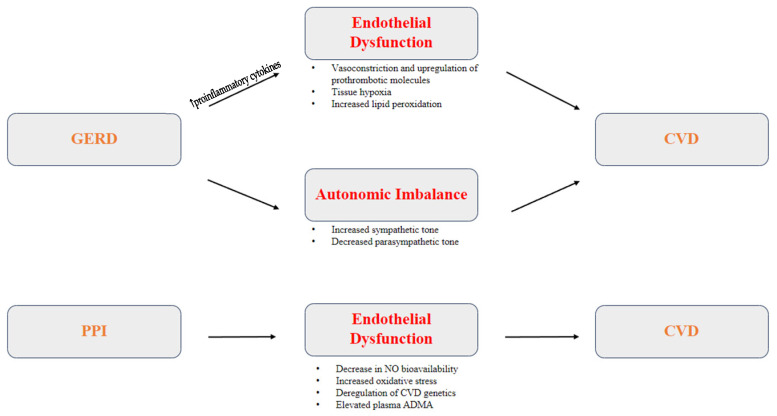
Proposed pathophysiology of GERD and PPI therapy as risk factors for CVD.

The direct pathophysiological connection between GERD and CVD has yet to be established, but various proposed mechanisms suggest a multifactorial process (Figure 2). Studies have suggested different inflammatory, autonomic, and thrombotic pathways that could link chronic GERD to the development of CVD.

#### 3.3.1. Inflammation and Endothelial Dysfunction

Endothelial dysfunction has been proposed as the primary mechanism in the pathogenesis of ischemic heart disease [21]. The endothelium is a vital semi-permeable barrier involved in hemostasis, thrombosis, and regulation of vascular tone [21]. It utilizes vasodilatory factors like nitric oxide (NO), prostacyclin (PGI2), and endothelium-derived hyperpolarization factor (EDHF), and vasoconstrictive factors like thromboxane A1 (TXA2) and endothelin-1 (ET-1) [21]. Chemical and mechanical stress on the endothelial cells induces a release of vasodilatory factors [21]. Dysfunction of the endothelium occurs when there is an inadequate production or bioavailability of endothelial NO, causing endothelial cells to become prothrombotic and proinflammatory [21]. This proinflammatory process can be potentiated by various underlying risk factors including hypertension, oxidized LDL, diabetes, and GERD, leading to increased production of proinflammatory cytokines such as interleukin-1 (IL-1), interleukin-6 (IL-6), and tumor necrosis factor-alpha (TNF-α) [21]. These, in turn, upregulate the expression of endothelial E-selectin, vascular cell adhesion molecule-1 (VCAM-1), and intercellular adhesion molecule 1 (ICAM-1) [21]. Biomarkers of inflammation like C-reactive protein (CRP), fibrinogen, and serum amyloid A (SSA) can be elevated through various phases of endothelial dysfunction [21,22,23,24].

A prospective study by Oparin et al. in 2017 aimed to investigate the pathogenesis of endothelial dysfunction in patients with both ischemic heart disease (IHD) and GERD [25]. Endothelial functional assessments were performed on 105 adult patients divided between three groups [25]. Group 1 consisted of patients with both IHD and GERD, group 2 patients with GERD alone, and group 3 served as controls [25]. Measurements of ET-1, NO metabolites, and lipid peroxidation were obtained. Regional blood flow circulation was also assessed by measurement of blood flow velocity and celiac trunk circumference with sonography [25]. The results indicated that patients with IHD and concomitant GERD, as well as those with GERD alone, exhibited statistically significant increases in ET-1 and decreases in NO metabolites compared to controls [25]. However, those with IHD and GERD showed a significantly greater difference [25]. Patients with GERD alone also displayed a distinct reduction in celiac trunk diameter and blood flow velocity in comparison with control groups, leading to reduced tissue oxygenation and resultant increased lipid peroxidation products [25]. It can be inferred from this data that GERD may contribute to the endothelial proinflammatory process through increased lipid peroxidation and tissue hypoxia.

#### 3.3.2. Autonomic Imbalance

Autonomic imbalance, characterized by an overactive sympathetic system and underactive parasympathetic system, has been associated with various pathological conditions, including cardiovascular disease [26]. Numerous studies have demonstrated autonomic nervous system disturbances in patients with GERD, including supraventricular and ventricular arrhythmias [27,28,29,30]. A study by Tougas et al. in 2001 compared autonomic tone between acid-sensitive patients, such as those with GERD, to acid-insensitive patients using power spectral analysis of heart rate variability before and after esophageal acidification [31]. Their results found no significant manometric changes during acidification in either group [31]. Acid-sensitive patients were found to be more likely to exhibit decreased resting vagal activity with higher resting heart rate and increased vagal cardiac outflow during acid infusion compared to acid-insensitive patients and healthy controls [31]. The loss of vagal tone in response to excess gastric acid in GERD patients may suggest an alternative pathway by which GERD may predispose patients to CVD [31].

### 3.4. Diagnosing GERD

GERD is a prevalent clinical condition diagnosed and managed by gastroenterologists, surgeons, and primary care providers. The American College of Gastroenterology (ACG) defines GERD as “the condition in which reflux of gastric contents into the esophagus results in symptoms and/or complications [32,33]. It is objectively defined by the presence of characteristic mucosal injury seen on endoscopy and/or abnormal esophageal acid exposure demonstrated on a reflux monitoring study” [32,33].

Classical GERD symptoms include heartburn and regurgitation, with heartburn being the most common symptom [32,33]. It is subjectively described as a burning sensation beneath the sternum that can radiate from the epigastrium towards the neck, often accompanied by an acid or bitter taste in the mouth [32,33]. However, these symptoms are often nonspecific and can be attributed to other esophageal conditions, such as eosinophilic esophagitis, cardiac or pulmonary disease, hernias, and more. Atypical extraesophageal symptoms can include chronic cough, dysphonia, asthma, sinusitis, laryngitis, and dental erosions, but they have poor sensitivity and specificity for the diagnosis of GERD [32,33].

**Figure 3:**
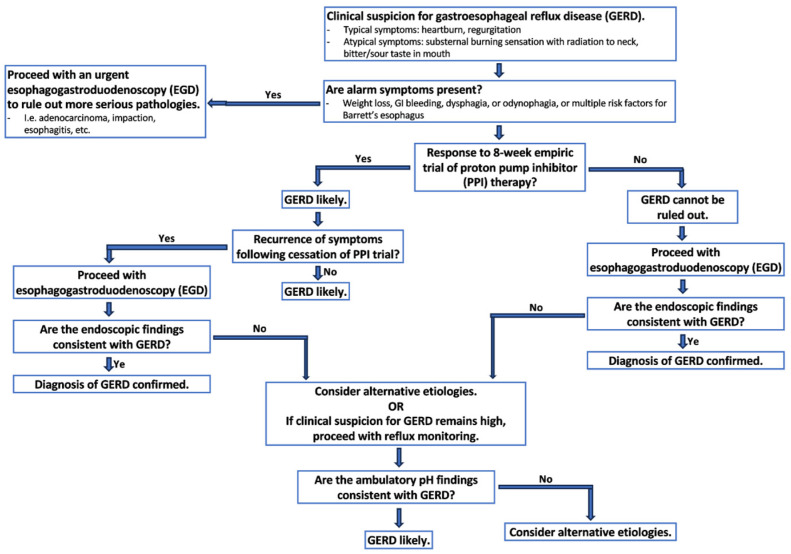
Proposed diagnostic algorithm for GERD.

There is no gold standard method for GERD diagnosis [32,33]. Instead, the diagnosis should rely on clinical history, endoscopic findings, reflux monitoring, and response to medical or surgical intervention (Figure 3) [32,33]. An 8-week empiric trial of proton pump inhibitors (PPIs) is recommended if classical symptoms are present followed by discontinuation of PPIs to monitor for symptom recurrence [32,33]. A diagnostic endoscopy is recommended within 2–4 weeks if the 8-week PPI trial fails or if symptoms reoccur [32,33]. The presence of alarm symptoms, such as significant weight loss, GI bleeding, dysphagia, or odynophagia, or multiple risk factors for Barrett’s esophagus should prompt an endoscopy as the first-line diagnostic modality given concern for more serious alternative pathologies, such as adenocarcinoma, esophagitis, or others [32]. In patients in which the diagnosis of GERD is suspected but endoscopic findings are inconsistent with GERD, reflux monitoring can be performed off PPI therapy to appropriately diagnose or exclude [32,33]. Ambulatory reflux monitoring off therapy is not recommended as the sole diagnostic test for GERD in patients with established endoscopic evidence of reflux esophagitis or in patients with long-segment Barrett’s esophagus [32,33].

### 3.5. Management of GERD and Its Relation to CVD

#### 3.5.1. Lifestyle Modifications

Lifestyle modifications, when combined with medical therapy, are effective in reducing GERD symptoms [32,33]. They should focus on mitigating the 14 shared risk factors between CVD and GERD as established by Sun et al., including BMI, HDL-C, triglyceride levels, type 2 diabetes mellitus, insomnia, and depression [15]. Common lifestyle interventions could include weight loss for overweight patients, sleeping with the head of the bed elevated, cessation of tobacco and alcohol use, avoidance of meals and bedtime snacks, eating with an upright posture, and avoidance of potentially aggravating foods, including coffee, chocolate, carbonated beverages, spicy foods, acidic foods, and foods with high fat content [32,33]. It is important to note that, while lifestyle modifications are commonly recommended, there is limited supporting evidence of their efficacy [32,33]. The available studies are often small scale and lack control groups, making interpretation challenging [32,33].

Weight loss has also been positively correlated with reduced prevalence of reflux symptoms in several studies, including randomized control trials and prospective cohort studies. One randomized control trial by Singh et al. in 2013 demonstrated a significant correlation between percent body weight loss and reduction in GERD symptoms [34]. Of their 332 cohort of overweight/obese patients, 97% of patients lost weight with an average of 13 ± 7.7 kg lost and 81% had a reduction in GERD symptom scores with 65% having a complete resolution and 15% with partial resolution [34]. The Nurses’ Health Study in 2006 further corroborates this association using logistic regression analysis to show a dose-dependent relationship between increasing body mass index (BMI) and the frequency of reflux symptoms in their nearly 10,600 patient cohort [35]. The HUNT study by Ness-Jensen et al. in 2013 was a prospective population-based cohort study consisting of approximately 29,600 patients again found that weight loss was dose-dependently associated with a reduction in GERD symptoms and increased treatment success when combined with medical management of GERD [36].

The HUNT study from 2013 also demonstrated a positive correlation between smoking cessation and improvement in GERD symptoms [37]. Of the approximately 29,600 participants, daily tobacco smoking cessation among individuals with normal BMI and taking antireflux medication at least weekly reduced GERD symptoms (adjusted OR 1.78, 95% CI 1.07–2.97) compared with persistent daily smoking [37]. However, no association was appreciated in participants with mild GERD symptoms or less frequent antireflux medication use [37]. This study suggests that, while tobacco smoking may be a contributor to GERD, obesity may be the predominant factor in obese patients. Despite its apparent potentially minor effect on GERD symptomatology, smoking cessation should still be encouraged amongst patients with GERD and CVD [38]. A prospective cohort study by Kohata et al. evaluated the effects of smoking cessation on GERD symptoms and health-related quality-of-life at 1-year post-cessation and found that nearly half of the participants who achieved smoking cessation experienced improved GERD symptoms and health-related quality of life compared to only 20% of those who failed to achieve smoking cessation [39].

Coincidentally, lifestyle changes implemented for the sake of reducing GERD symptoms can also mitigate the onset or progression risk of CVD. Diets consisting of high fat, low fiber, and low fruit and vegetable meal choices have been widely associated with adverse cardiovascular events, as opposed to the Mediterranean diet, which is rich in fruits, vegetables, and healthy fats and oils, has been associated with reduced risk of these events [40]. The Spanish CORDIOPREV trial by Delgado-Lista et al. in 2022 was a long-term randomized controlled trial that randomly assigned 1000 participants to either a Mediterranean diet or low-fat diet intervention groups at a 1:1 ratio [40]. At 7-year follow-up, the major adverse cardiovascular events (AMI, revascularization, ischemic stroke, peripheral artery disease, and cardiovascular death) were significantly lower in patients who were assigned to the Mediterranean diet compared to low-fat diet alone (multi-adjusted HR 0.64, 95% CI 0.489–0.915), suggesting secondary prevention of adverse cardiovascular events with the Mediterranean diet is superior to a low-fat diet alone [40]. Adherence to the Mediterranean diet has also been demonstrated to reduce the occurrence of GERD in an Albanian cross-sectional study by Mone et al. in 2016 [41]. In their cohort, 817 patients were categorized into largely Mediterranean diet-consuming versus largely non-Mediterranean diet-consuming groups [41]. Irrespective of demographic and socioeconomic characteristics and lifestyle factors including eating habits, largely non-Mediterranean diet consumption was positively correlated to GERD disease risk (fully adjusted OR 2.3, 95% CI 1.2–4.5), suggesting that adoption of the Mediterranean diet can be beneficial in the management GERD [41].

Many studies have shown that smoking cessation has been significantly associated with a reduction in CVD risk [42]. A 2023 systematic review by Parmar et al. pooled the results from twenty studies that compared smoking to adverse cardiovascular outcomes [42]. Exposure to tobacco smoke at low levels was associated with a steep rise in cardiovascular risk, however, the dose–response relationship was less profound as exposure increased [42]. Nevertheless, smoking cessation should be encouraged, especially in those with or at-risk for CVD or GERD.

#### 3.5.2. Medical Therapy

Acid suppressive therapy remains the mainstay of treatment for GERD. Several different agents are available, including antacids, histamine 2 receptor antagonists (H2RA), and proton pump inhibitors (PPIs). Antacids are primarily used as rescue therapy due to their availability over the counter, whereas PPIs and H2RAs are used for chronic suppressive therapy. PPIs are the most commonly prescribed medication for GERD due to their consistently superior efficacy in relieving heartburn and regurgitation, as well as promoting healing as compared to H2RAs [43].

PPIs work by inhibiting the hydrogen-potassium ATPase enzyme, also known as the proton pump, in the stomach’s parietal cells [44]. This enzyme is the final step in the secretion of stomach acid [44,45]. PPIs are ingested in a prodrug form and activated through acid-catalyzed cleavage in the acid secretory canaliculi of the parietal cells [44]. They are subsequently metabolized by hepatic P450 enzymes, which can be variable among the population, explaining why different patients have variable responses to select PPIs [44].

Histamine release from enterochromaffin-like cells and mast cells in the corpus gastric glands normally directly binds histamine receptors on gastric parietal cells and stimulates the release of acid [45]. H2RAs work to reduce gastric acid secretion by competitively antagonizing histamine binding, resulting in reduced acid secretion [46]. Unlike PPIs, all H2RAs have similar efficacy in decreasing gastric acid secretion [33,46]. In cases of refractory GERD, especially those with nocturnal symptoms, concomitant administration of H2RAs with PPIs has been shown to increase gastric acid suppression [47].

PPIs and H2RAs are frequently prescribed to patients with both GERD and concomitant coronary artery disease or previous myocardial infarctions due to concerns about chronic excess gastric acid secretion with chronic NSAID, antiplatelet, and/or anticoagulant agents and the increased risk for upper gastrointestinal bleeding [48]. However, new data suggests that PPIs may play a role in the incidence, progression, and mortality of cardiovascular disease [49].

A recent umbrella review by Teperikidis et al. from 2023 analyzed seven systematic reviews and analyses, involving a total of 46 randomized controlled trials and 33 observational studies, to examine the association between PPI use and adverse cardiovascular outcomes, including stroke, AMI, and all-cause mortality [50]. While the results of individual studies were mixed, most studies that included observational data reported a positive association between PPI use and adverse cardiovascular outcomes [50].

Long-term use of PPIs has been shown to increase the risk of cardiovascular events through multiple pathways, including inhibition of lysosomal acidification, impairment of protein homeostasis, and reduction in telomere length, resulting in endothelial dysfunction and endothelial cell senescence [48]. A study by Taneja et al. found that subacute administration of PPIs did not produce any toxicological impact on the endothelial tissue, but subchronic administration of PPIs caused moderate vascular endothelial dysfunction in a dose-dependent manner, suggesting a decrease in NO bioavailability and increased oxidative stress may lead to vascular endothelial dysfunction [51]. Another study by Hamzeloo-Moghadam et al. identified several critical genes associated with cardiovascular disease (CTNNB1, HNRNPA1, SRSF4, TRA2A, SFPQ, and RBM5) that can be deregulated with PPI use [49]. Furthermore, several studies have shown that PPIs elevate plasma asymmetrical dimethylarginine (ADMA) levels, an endogenous inhibitor of NO synthase, through inhibition of dimethylarginine dimethylaminohydrolase, an enzyme that degrades ADMA [48,52].

In the context of AMI or situations requiring antiplatelet agents like clopidogrel, there is concern that PPIs may impair the metabolic activation of these antiplatelet agents, increasing the risk of adverse cardiac events [53]. Bioactivation of clopidogrel by CYP2C19 may be reduced due to competitive inhibition with some PPIs, such as omeprazole [53]. While other PPIs, like pantoprazole, are not metabolized by this enzyme, studies have shown that they also have similar adverse cardiovascular outcomes, suggesting a mechanism other than prodrug activation may be contributing [54].

Several studies have also suggested that the use of PPIs may independently be associated with an increased risk of AMI. A case-control study by Shih et al. in 2014 found that PPI use was associated with a 1.58-fold greater risk of recurrent myocardial infarction compared to controls [55]. Another case-control study by Valkhoff et al. in 2011 demonstrated that current PPI use among clopidogrel users was associated with an increased risk of recurrent myocardial infarction compared to no PPI use (OR 1.62, 95% CI 1.15–2.27), but not when compared to past PPI use (OR 0.95, 95% CI 0.38–2.41) [56]. Extrapolation of these data suggests that PPI usage alone might increase the risk of recurrent MI in patients and should be avoided in patients with a history of AMI.

Two studies have argued that inappropriate use of PPIs has unnecessarily increased the risk of adverse cardiovascular outcomes. Hu et al. in 2022 and Ma et al. in 2022 both reported an increased ASCVD risk associated with GERD, especially in patients without indications for the medications [35,47,48,57].

Conversely, the COGENT trial by Bhatt et al. in 2010, an international, randomized, double-blind, double-dummy, placebo-controlled, parallel-group trial, compared the adverse gastrointestinal and cardiovascular events in a randomized cohort of patients receiving fixed-dose clopidogrel and aspirin plus omeprazole to those receiving clopidogrel and aspirin alone [58]. A total of 3861 patients were included in the analyses [58]. The results demonstrated that prophylactic use of PPIs reduced the rate of upper gastrointestinal bleeding (HR 0.13, 95% CI 0.03–0.56, *p* = 0.001) compared to placebo; however, no significant difference in cardiovascular events was noted between the groups (HR with omeprazole 0.99, 95% CI 0.68–1.44, *p* = 0.96) [58].

Due to the concerns about antiplatelet attenuation with PPIs and the increased risk of worse cardiovascular outcomes, many clinicians have explored the use of H2RAs for patients on dual antiplatelet therapy [59]. A study in 2014 by Luo et al. found that H2-receptor activation exaggerated myocardial injury by promoting myocardial mitochondrial dysfunction and by increasing cardiac vascular endothelial permeability [60]. This information suggests that the use of H2RAs in patients with known coronary atherosclerosis or following AMIs may lead to improved outcomes compared to PPI use; however, further studies should be conducted to investigate this hypothesis. Recent studies have also suggested that H2RAs are non-inferior to PPIs when combined with dual antiplatelet therapy for major adverse cardiovascular outcomes and may even have utility in heart failure. Given the known adverse cardiovascular effects of PPIs and interaction with clopidogrel, further consideration and studies could investigate the use of H2RAs in these cases [43].

#### 3.5.3. Mechanical Interventions

Surgical management of pre-existing conditions that could potentiate reflux disease, such as hiatal hernias, should be performed. In those without pre-existing contributors, mechanical interventions may be considered in the appropriate patients with truly refractory GERD. However, the diagnosis and management of GERD refractory of antireflux agents and lifestyle modifications poses a significant challenge to clinicians. A randomized controlled trial by Spechler et al. in 2019 identified 366 patients from Veterans Affairs gastroenterology clinics referred to clinic for refractory GERD [61]. During the pre-randomization period, participants underwent a two-week PPI trial and those with persistent symptoms underwent endoscopy, esophageal biopsy, esophageal manometry, and multichannel intraluminal impedance-pH monitoring [61]. Ultimately, 42 patients were excluded due to positive response to a two-week trial of PPI therapy, 23 patients were excluded due to non-GERD esophageal disorders causing GERD-mimicking symptoms, and 99 patients were excluded due to functional heartburn (not related to GERD or other histopathological, motility, or structural abnormality) producing symptoms [61]. Only 78 patients were able to be randomized into study groups [61].

Once patients have demonstrated true refractoriness to antireflux medications and lifestyle interventions, they may be considered for mechanical interventions. Some studies have supported mechanical interventions as superior to medical management in refractory cases [61,62,63,64]. Proposed interventions include surgical fundoplication, magnet therapy, and endoscopic interventions.

Fundoplication is a surgical technique that recreates the lower esophageal sphincter pressure by wrapping the fundus of the stomach around the esophagus. It is often performed laparoscopically due to improved morbidity and shorter hospital length of stays compared to open approaches. Clinician preference drives the use of different techniques, including Dor fundoplication (an anterior 180-degree wrap), Toupe fundoplication (a posterior 270-degree wrap), and Nissen fundoplication (a total posterior 360-degree wrap) [65]. Due to the high mechanical stress at the gastro-esophageal junction, the fundoplication may fail overtime or may even be too tight, provoking dysphagia, but often has a high post-operative patient satisfaction rate [66,67].

The previously introduced study by Spechler et al. randomly assigned 78 patients with truly refractory GERD to either surgical intervention with laparoscopic Nissen fundoplication or continued medical management [61]. At 12-month follow-up, the incidence of treatment success with surgery was significantly superior to medical management with 67% of surgical patients responding to the intervention compared to 28% in the medical management group (*p* = 0.007, Hochberg-adjusted significance threshold, 0.025) and 12% in the control medical group (*p* < 0.001; Hochberg-adjusted significance threshold, 0.017) [61]. During follow-up evaluation approximately 10.6 years from their respective interventions, 92% of patients in the medical management group and 62% in the surgical intervention group reported continuation of antireflux medications following completion of the original study (*p* < 0.001) [66]. Symptom scores were significantly lower in the surgical treatment group following a trial of antireflux-free medication, but there were no observed significant differences observed in the endoscopic grade of esophagitis between the groups (*p* = 0.03) [66]. Interestingly, heart disease was the cause of death in more patients who received surgical intervention compared to medical management by a statistically significant margin (448% vs. 20%, *p* = 0.004) [66].

A randomized control trial by Rudolph-Stringer et al. in 2022 compared the 15-year efficacy between laparoscopic Nissen to Dor fundoplication in a cohort of 107 participants [67]. After 15–20 years, both techniques achieved similar success (mean satisfaction score 8.4 vs. 8.0, *p* = 0.444; 86.8% vs. 90.2% satisfied with outcome), but Dor fundoplication was associated with higher rates of heartburn (mean score 3.2 vs. 1.4, *p* = 0.001) and PPI use (41.7% vs. 17.1%, *p* = 0.023) compared to Nissen fundoplication [67]. However, less dysphagia to solids (mean score 1.8 vs. 3.3, *p* = 0.015) and better ability to belch (84.2% vs. 65.9%, *p* = 0.030) were better with Dor fundoplication [67]. Overall, Nissen fundoplication was suggested to produce better reflux symptom control with an increased rate of adverse side effects.

A prospective study by Zornig et al. in 2002 compared Nissen to Toupe fundoplication using a cohort of 100 participants at a 1:1 ratio [68]. After 4-month follow-up, the findings suggested increased patient satisfaction in 88% of Nissen fundoplication participants and 90% in those who underwent the Toupe technique [68]. However, post-operative dysphagia was present in more participants who received Nissan fundoplications compared to Toupe (30 vs. 11, *p* < 0.001), suggesting that the Toupe technique may be superior to Nissan based on a superior adverse effect rate profile [68].

Another laparoscopic procedure known as magnetic sphincter augmentation (MSA) by the Linx Reflux Management System has also shown promise in managing refractory reflux disease [62,69]. It involves implantation of a small device comprising interlinked titanium beads with magnetic cores around the lower esophageal sphincter, augmenting its function [69]. A randomized controlled trial by Bell et al. in 2020 compared MSA to PPI therapy in a cohort of 152 patients with moderate to severe GERD symptoms [62]. After 1-year follow-up, 61 of the 75 patients who were treated with MSA (81%) had improvements in GERD health-related quality of life scores and 68 patients (91%) discontinued daily PPI use [62]. A total of 48 of 69 patients who underwent pH evaluation after 1-year follow-up were found to have pH normalization [62]. Furthermore, no peri-operative events, device explants, erosions, or migrations were noted [62]. This study suggests MSA is superior to PPI-only therapy in this patient population.

Long-term outcomes of MSA have also been favorable [70,71]. A prospective study by Ferrari et al. in 2020 followed 335 patients who underwent MSA implantation for 6 to 12 years following their procedures [70]. The results showed significant improvement in mean total GERD-specific health-related quality of life scores from 19.9 to 4.01 (*p* < 0.001) with discontinuation of PPIs in 79% of patients [70]. The mean total percent time with pH < 4 decreased from 9.6% at baseline to 4.1% (*p* < 0.001) with 89% of patients achieving normalization [70].

Endoscopic intervention for patients with refractory GERD has also produced promising results. Two of the more commonly utilized endoscopic techniques include radiofrequency energy treatment (Stretta) and transoral incisionless fundoplication (TIF) [63,72].

The Stretta procedure usually uses radiofrequency energy, which is applied to the muscles of the lower esophageal sphincter and the gastric cardia using four needle electrodes that extend out from the balloon catheter into the muscle at six levels across the gastroesophageal junction, resulting in an improvement of reflux symptoms [5]. Stretta is ideal for patients who have contraindications to medical therapy or do not qualify or refuse surgical intervention [72].

A small randomized prospective study by Kalapala et al. assigned 20 patients to Stretta intervention or the control group with PPI therapy only [73]. Only 3 months after Stretta intervention, 80% of patients reported an improvement in quality of life compared to only 40% in the control group. A significant improvement in GERD symptoms were reported (*p* < 0.05) with 60% of these patients reporting PPI discontinuation compared to no change in the control group [73].

Transoral incisionless fundoplication (TIF) is performed by creating full-thickness serosa-to-serosa plications and reconstruct valves approximately 3 cm in length and 270 to 300 degrees in circumference [72]. The gastric fundus is then folded, wrapped around and fastened to the distal esophagus, as seen in Nissen fundoplication procedures [72]. A randomized controlled trial by Kalapala et al. in 2021 compared 35 patients who underwent TIF to 35 patients who underwent a sham procedure [64]. The results suggested that those who underwent TIF experienced a statistically significant improved quality of life at 8-to-9-month follow-up (*p* < 0.001) [64]. A total of 62.8% of patients who underwent TIF had discontinued PPI therapy at 12-month follow-up compared to 11.4% in the control group (*p* < 0.001) [64].

These results were corroborated by another randomized controlled trial called the TEMPO trial by Trad et al. from 2013 through 2018 [74]. A total of 63 patients were randomly assigned to the TIF group or PPI group [74]. All patients in the PPI group elected for crossover to the TIF group at 6-month follow up [74]. Of the patients, 60 were available for evaluation at 1-year, 52 at 3-year, and 44 at 5-year follow-ups [74]. Troublesome regurgitation and atypical symptoms were eliminated in greater than 86% and 80% of patients at the end of the 5-year follow-up period, respectively [74]. PPI use was reduced from 100% at baseline to only 34% at 5-year follow-up [74].

Another randomized controlled trial by Kaindlstorfer et al. in 2013 compared TIF to laparoscopic antireflux surgery (Nissen or Toupe) in 70 adult patients [75]. The results indicated that reflux events were reduced in both groups, but reductions in reflux-related esophageal acid scores measured via 24 h impendence pH monitoring were only significant in the antireflux surgery group [75]. The adverse side effect profile favored TIF over Nissen antireflux surgeries [75]. This study suggests TIF intervention may be superior to surgical interventions due to similar success rates and less adverse side effects.

## 4. Future Direction of Studies

Given the growing concern that GERD could be a risk factor for the development of CVD, future studies should be directed toward a better understanding of the causative impact of GERD and management of GERD on CVD. The impact of lifestyle modifications on GERD management and cardiovascular risk reduction should be further explored, even though healthy lifestyle recommendations are commonly employed. Future studies should aim to better understand the mechanisms underlying the adverse effects associated with PPI use and cardiovascular disease. Studies could investigate whether specific PPIs have varying impacts on cardiovascular outcomes and explore the genetic factors that influence individual responses to PPI therapy. Guidelines for PPI prescription in CVD, especially with long-term use, should be established. More studies should evaluate the safety and efficacy of alternative medications for GERD, such as H2RAs in patients with GERD and those requiring antiplatelet therapy to determine whether they present a safer option. The potential benefits and risks of using H2RAs in combination with antiplatelet agents should be explored in greater detail. Furthermore, with increasing success rates of mechanical interventions cases of refractory GERD, more research needs to be performed to evaluate the impact of these interventions on the onset, progression, and adverse outcomes with CVD, especially considering the unexpected increased rate of death due to cardiovascular disease with Nissen fundoplication appreciated in the Spechler et al. trial.

## 5. Conclusions

In conclusion, the relationship between GERD and cardiovascular disease is a complex and emerging area of research. Recent studies have hypothesized potential connections between these two prevalent conditions, suggesting that GERD may serve as a risk factor for the onset and progression of cardiovascular disease through various pathophysiological pathways including endothelial dysfunction and autonomic imbalance.

The management of GERD, primarily with H2RAs and PPIs, not only offers relief from GERD symptoms but may also potentially reduce the incidence and progression of CVD. However, this promising approach must be balanced with the potential cardiovascular risks associated with these medications. Prudent prescribing practices and a careful evaluation of each patient’s needs are crucial to prevent adverse cardiovascular events.

Future large-scale prospective studies are necessary to establish the causative impact of GERD on cardiovascular disease and help create evidence-based guidelines for its management with respect to its cardiovascular implications. The evolving understanding of the complex interplay between GERD and cardiovascular disease offers exciting prospects for improving patient care and reducing the burden of both conditions on a global scale.

## Figures and Tables

**Table 1:** Summary of notable studies related to GERD and CVD including PPIs and CVD.

Year	Authors	Title	Study Type	Conclusions
2023	Chen J et al.	Risk of incident cardiovascular disease among patients with gastrointestinal disorder: prospective cohort study of 340,862 individuals	Prospective cohort study	Any gastrointestinal disease was associated with an elevated risk of overall CVD (HR 1.38, 95% CI 1.35–1.42). GERD was associated with CVD (HR 1.41, 95% CI 1.35–1.46), with a stronger correlation in women, patients with a body mass index ≥ 25 kg/m^2^, and patients ≤ 60 years old.
2023	Geng T et al.	Proton pump inhibitor use and risks of cardiovascular disease and mortality in patients with type 2 diabetes	Population-based cohort	PPI use is associated with a higher risk of CVD events and mortality among patients with type 2 diabetes mellitus.
2023	Sun L et al.	Helicobacter pylori infection and risk of cardiovascular disease	Meta-analysis	Helicobacter pylori infection is associated with a mildly increased risk of CVD.
2023	Teperikidis E et al.	Does the long-term administration of proton pump inhibitors increase the risk of adverse cardiovascular outcomes? A ChatGPT powered umbrella review	Umbrella review	A causal relationship between PPI use and an increased risk of MACE cannot be ruled out.
2022	Song J et al.	Association between gastroesophageal reflux disease and coronary atherosclerosis	Retrospective cohort study	GERD was associated with higher degrees of coronary atherosclerosis by CACS (*p* = 0.008) but did not increase the risk of a higher CACS (OR = 1.018, 95% CI 0.865–1.198).
2022	Sun X et al.	A Mendelian randomization study to assess the genetic liability of gastroesophageal reflux disease for cardiovascular diseases and risk factors	Mendelian randomization study	GERD was associated with 7 CVD outcomes and 9 cardiovascular risk factors.
2022	Ma Y et al.	Acid suppressants use and risk of atherosclerotic cardiovascular disease in middle-aged and older adults	Prospective cohort study	PPI use is associated with increased risk of ASCVD, particularly amongst participants without indications for medications.
2022	Maret-Ouda et al.	Proton pump inhibitor and clopidogrel use after percutaneous coronary intervention and risk of major cardiovascular events [3]	Retrospective cohort study	In patients who receive clopidogrel after PCI, concomitant use of PPI may increase the risk of major cardiovascular events.
2021	Bell E et al.	Association of proton pump inhibitors with higher risk of cardiovascular disease and heart failure	Prospective cohort study	Long-term PPI use was associated with twice the risk of total CVD and HF compared with nonusers.
2021	Rooney M et al.	Proton pump inhibitor use, hypomagnesemia and risk of cardiovascular diseases: The atherosclerosis risk in communities (ARIC) study	Prospective cohort study	PPI users had a higher prevalence of hypomagnesemia than nonusers. PPI users also had higher CVD risk than nonusers; however, it appears unlikely that hypomagnesemia explains associations of PPIs with CVD risk.
2020	Eisa M et al.	The risk of acute myocardial infarction in patients with gastroesophageal reflux disease	Observational	GERD is a risk factor for AMI, higher than male gender and obesity.
2020	Wang B et al.	A meta-analysis of the association between helicobacter pylori infection and risk of atherosclerotic cardiovascular disease.	Meta-analysis	H pylori infection increases the risk of adverse cardiovascular events by 51%, with an even greater effect on AMI (OR = 1.80, 95% CI 1.42–2.26) and cerebrovascular disease (OR = 1.54, 95% CI 1.27–1.89).
2019	Teragawa H et al.	History of gastroesophageal reflux disease in patients with suspected coronary artery disease	Experimental	The presence of GERD may increase the incidence of vasospastic angina in patients with suspected coronary artery disease (OR 7.8; *p* < 0.01).
2019	Khomenko et al.	Features of endothelial dysfunction in elderly persons with coronary heart disease and concomitant gastroesophageal reflux disease [4]	Experimental	Endothelial dysfunction manifests itself as a decrease in stable nitric oxide metabolite levels and an increase in endothelin-1 levels, disturbance of celiac trunk regional blood flow, causing a decrease in esophageal tissue resistance, leading to lower esophageal sphincter dysfunction
2018	Landi et al.	No increase in risk of acute myocardial infarction in privately insured adults prescribed proton pump inhibitors vs. histamine-2 receptor antagonists (2002–2014) [5]	Retrospective cohort study	No difference in increased risk of AMIs with PPIs versus H2RAs.
2018	Nguyen et al.	No significant association between proton pump inhibitor use and risk of stroke after adjustment for lifestyle factors and indication [6]	Retrospective Cohort Study	No significant association between PPI use and ischemic stroke, after accounting for indications for PPI use. Prior reports of an increased risk of stroke may be due to residual confounding related to chronic conditions associated with PPI use.
2017	Oparin et al.	The role of endothelial dysfunction in the mechanism of gastroesophageal reflux disease development in patients with ischemic heart disease	Experimental	In patients with ischemic heart disease and concomitant GERD, endothelial dysfunction manifested by a significant increase in the levels of endothelin-1 and lipid peroxidation products, with decreased levels of nitric oxide metabolites, regional blood flow and quality of life.
2017	Lei W et al.	Risk of acute myocardial infarction in patients with gastroesophageal reflux disease: A nationwide population-based study	Prospective cohort study	GERD was associated with a higher risk of developing an AMI compared to controls (HR = 1.48; 95% CI: 1.31–1.66, *p* < 0.001).
2016	Chen C et al.	Association between gastroesophageal reflux disease and coronary heart disease	Population-based cohort	GERD was associated with a higher risk of developing coronary heart disease compared to controls (aHR = 1.67, 95% CI = 1.34–2.08) and in patients with GERD who were treated with PPI therapy for more than 1 year compared to those treated for less than 1 year (aHR = 1.56, 95% CI = 1.39–1.74).
2015	Shah et al.	Proton pump inhibitor usage and the risk of myocardial infarction in the general population [7]	Systematic review	GERD patients exposed to PPIs have a 1.16 fold increased risk of AMI, regardless of clopidogrel use.
2014	Shih C et al.	Proton pump inhibitor use represents an independent risk factor for myocardial infarction	Propensity-score matched study case-crossover study	Use of PPIs may be independently associated with an increased risk of MI. However, the benefits of PPIs may greatly outweigh the risks of adverse cardiovascular effects, with number needed to harm of 4357.
2014	Unal et al.	The effects of proton pump inhibitors on the development of post-stenting major adverse cardiovascular events in patients with acute coronary syndrome	Prospective cohort study	ADMA and copeptin levels may be significantly increased in patients started on imminent DAPT and PPI therapy after PCI.
2013	Ghebremariam Y et al.	Unexpected effect of proton pump inhibitors	Observational	Biochemical, cellular, ex vivo, and in vivo data revealing that PPIs directly interact with and significantly inhibit human DDAH activity, thereby increasing endothelial and serum ADMA levels. The increase in ADMA levels would be anticipated to impair vascular NOS activity, to increase oxidative stress, to reduce vasodilator function, and to impair vasoprotective mechanisms.
2013	Luo T et al.	Histamine H2 receptor activation exacerbates myocardial ischemia/reperfusion injury by disturbing mitochondrial and endothelial function	Experimental	H2R activation exaggerates myocardial I/R injury by promoting myocardial mitochondrial dysfunction and by increasing cardiac vascular endothelial permeability.
2013	Liu et al.	Acid reflux in patients with coronary artery disease and refractory chest pain [8]	Prospective cohort study	Refractory chest pain in patients with CAD can be partially noncardiac chest pain (NCCP) secondary to acid reflux. The combined use of common cardiac drugs may predispose or aggravate GERD. Short-term proton pump inhibitor (PPI) therapy not only restores a normal esophageal pH, but also significantly improves the general health-related quality of life (HRQL) of patients.
2012	Schmidt et al.	Concomitant use of clopidogrel and proton pump inhibitors is not associated with major adverse cardiovascular events following coronary stent implantation [9]	Retrospective cohort study	The use of PPIs as a class did not modify the protective effect of clopidogrel, but its use was associated with major adverse cardiovascular events itself, particularly among patients having used PPIs before percutaneous coronary intervention.
2010	Gupta et al.	Risk of adverse clinical outcomes with concomitant use of clopidogrel and proton pump inhibitors following percutaneous coronary intervention [10]		Concomitant use of clopidogrel and PPI in post-PCI patients is associated with a higher risk of MACE.
2008	Jansson C et al.	Severe symptoms of gastro-oesophageal reflux disease are associated with cardiovascular disease and other gastrointestinal symptoms, but not diabetes: a population-based study	Population-based, cross-sectional, case-control study	Myocardial infarction, angina pectoris, stroke and symptoms of nausea, diarrhea and constipation are associated with GERD.
2001	Tougas et al.	Cardiac autonomic function and oesophageal acid sensitivity in patients with non-cardiac chest pain	Experimental	Patients with angina-like pain during direct esophageal acidification have decreased resting vagal activity.

## Data Availability

No new data was created or analyzed in this study.
